# Analysis of the Microstructure and Mechanical Performance of Resistance Spot-Welding of Ti6Al4V to DP600 Steel Using Copper/Gold Cold-Sprayed Interlayers

**DOI:** 10.3390/ma17133251

**Published:** 2024-07-02

**Authors:** Krzysztof Szwajka, Joanna Zielińska-Szwajka, Marek Szewczyk, Marwan T. Mezher, Tomasz Trzepieciński

**Affiliations:** 1 Department of Integrated Design and Tribology Systems, Faculty of Mechanics and Technology, Rzeszow University of Technology, ul. Kwiatkowskiego 4, 37-450 Stalowa Wola, Poland; kszwajka@prz.edu.pl (K.S.); m.szewczyk@prz.edu.pl (M.S.); 2 Department of Component Manufacturing and Production Organization, Faculty of Mechanics and Technology, Rzeszow University of Technology, ul. Kwiatkowskiego 4, 37-450 Stalowa Wola, Poland; j.zielinska@prz.edu.pl; 3 Departamento de Deseño na Enxeñaría, Universidade de Vigo, 36310 Vigo, Spain; marwantahir90@gmail.com; 4 Institute of Applied Arts, Middle Technical University, Baghdad 10074, Iraq; 5 Department of Manufacturing Processes and Production Engineering, Rzeszow University of Technology, al. Powstancow Warszawy 8, 35-959 Rzeszow, Poland

**Keywords:** resistance spot-welding, titanium alloy (Ti6Al4V), DP600 steel, Cu interlayer, Au interlayer

## Abstract

In this article, an attempt was made to join DP600 steel and Ti6Al4V titanium alloy sheets by resistance spot-welding (RSW) using an interlayer in the form of Cu and Au layers fabricated through the cold-spraying process. The welded joints obtained by RSW without an interlayer were also considered. The influence of Cu and Au as an interlayer on the resulting microstructure as well as mechanical properties (shear force and microhardness) of the joints were determined. A typical type of failure of Ti6Al4V/DP600 joints produced without the use of an interlayer is brittle fracture. The microstructure of the resulting joint consisted mainly of the intermetallic phases FeTi and Fe_2_Ti. The microstructure of the Ti6Al4V/Au/DP600 joint contained the intermetallic phases Ti_3_Au, TiAu, and TiAu4. The intermetallic phases TiCu and FeCu were found in the microstructure of the Ti6Al4V/Cu/DP600 joint. The maximum tensile/shear stress was 109.46 MPa, which is more than three times higher than for a welded joint fabricated without the use of Cu or Au interlayers. It has been observed that some alloying elements, such as Fe, can lower the martensitic transformation temperature, and some, such as Au, can increase the martensitic transformation temperature.

## 1. Introduction

The resistance spot-welding (RSW) process involves joining metal elements together through a process in which contacting metal surface points are joined by the heat obtained from resistance to electric current [1,2,3]. Two metals can connect to each other because heat is released in the welding zone under the influence of current flow between the electrodes. As a result, the metal melts and joins under thermoplastic conditions [4,5]. The spot-welding cycle consists of three successive stages [1,6]: (I) pressing the joined sheets with the electrodes of the welding machine, (II) heating the joined elements due to the flow of current through the joined materials, and (III) after the weld cools down, creation of a permanent connection as a result of the solidification process. Resistance spot-welding technology is used to join elements made of carbon and alloy steels and non-ferrous metals [7,8,9]. The thickness of the welded materials depends on the power of the welding machine and the type of welded material. When welding sheet metals, there are three basic welding parameters: current flow time, welding current, and electrode pressure force [1,10].

RSW technology is primarily used in the automotive industry [11,12], usually in robotic systems [13]. There are usually 4000–6000 spot-welds in one motor vehicle [14,15]. Resistance welding is also used in the production of responsible structural elements of agricultural machines, industrial fittings, and tools for metal and woodworking [14]. The industrial importance of resistance welding technology is also determined by the possibility of combining similar and dissimilar materials and materials with different coatings [16,17,18]. The automotive industry is dominated by steel sheets with zinc-based coatings, metal-alloy coatings, or an additional organic layer, e.g., ZnNi(Cr) coatings [14,19,20].

Joints of non-ferrous metal alloys, especially titanium and aluminum, are widely used in the aerospace and automotive industries. Over the years, many attempts have been made to join these materials through resistance spot-welding [21,22]. In automotive applications, Ti6Al4V alloy is most often combined with carbon and high-strength steels, which, due to their high strength, are used in crumple zones and responsible structural elements [23]. According to The Automotive and Transportation Market Research Report [24], high-strength and low-alloy steels (i.e., dual phase (DP) steels, Complex Phase (CP) steels, transformation-induced plasticity steels, High-Strength Low-Alloy (HSLA) steels, and many others) currently constitute up to 60% of modern car body structures. The use of titanium alloys in body panels used in the automotive industry is due to their energy-absorbing deformation behavior and high strength-to-weight ratio [25]. Investigations aimed at combining titanium alloys and high-strength steels in automotive applications are therefore desirable.

Research on the RSW of titanium alloys and HSLA steels is limited due to the different thermal-mechanical properties of these materials. As a result, the mechanical properties deteriorate locally, which in the case of incorrectly adjusted welding parameters, results in poor joint performance. The key is to obtain the desired microstructure in the weld by providing the appropriate amount of heat to the joint [26]. The RSW of DP steels is a challenging task due to formation during cooling of the brittle martensitic phase in the fusion zone [27].

Spark plasma sintering (SPS) technology is associated with the uniqueness of the heating mechanism when combining dissimilar functional-graded materials (FGMs). SPS is widely used for the fabrication of hard metals [28] and coatings [29], ceramics [30,31,32], and FGMs [33]. Shichalin et al. [34] proposed application of the SPS technology for obtaining layered functionally graded materials based on the joined metals Cr-Ti-Fe-Co-Ni-Cu. The results confirmed the potential of SPS technology for producing functionally graded coatings and FGM. Shichalin et al. [28] fabricated WC-Ni-Fe hard metal alloys via rapid SPS consolidation. The advantages of SPS technology and ongoing challenges in modeling the SPS of ceramic and metallic systems are discussed in Abedi et al. [35] and Kumar et al. [36].

The studies available in the literature on the dissimilar resistance spot-welding of titanium alloys and steels are mainly limited to stainless steels. Taufiqurrahman et al. [37] investigated the RSW of Ti6Al4V titanium alloy and stainless steel 316L with an EN AW-5754 alloy interlayer. It was concluded that the increase of holding time increased the tensile/shear strength of the joint by elimination of voids in the weld. In another article, Taufiqurrahman et al. [38] used the Taguchi approach to optimize the process parameters of the RSW of Ti6Al4V/316L joints with an EN AW-5754 aluminum alloy interlayer. The intermetallic compound layer was found on both Ti6Al4V/AW-5754 and AW-5754/316L interfaces due to the heat input causing a diffusion reaction. Tu et al. [39] studied the mechanical properties and interfacial microstructure of pure titanium–SUS304 stainless steel joints subject to RSW with an EN AW-5052 aluminum alloy insert. They found that the tensile/shear property of the Ti/SUS304 joints can be improved by using an EN AW-5052 alloy insert between commercially pure (CP) titanium and SUS304 steel sheets. Intermetallic compounds (IMCs) are formed when two different metals diffuse between each other, creating materials that are a combination of the two materials. The possibility of creating IMCs is mainly determined by the electron structure of the atoms of the constituent elements, the electron concentration, and the size of the atomic radii. The creation of new phases takes place as a result of the diffusion process, the speed of which depends on the temperature. Li et al. [40] joined CP titanium and stainless steel sheets with an interlayer of Nb. The Nb intermediate layer prevents the direct contact of joined sheets, directly inhibiting the adverse reaction of the interface. The tensile shear strength results showed that the intermediate Nb layer improves the performance of the joint. Yu et al. [41] proposed ultrasonic seam-assisted RSW for welding Q235 mild steel and TC4 titanium alloy with a Cu interlayer. They concluded that the pre-metallurgical bonding between the Cu interlayer and steel effectively reduced the number of defects, such as pores and cracks.

The incorporation of various interlayers between joined sheets enables the enhancement of mechanical and surface properties of RSW joints [42]. So far, the influence of various interlayer elements on the joint strength of titanium alloy/steel RSW joints has not been thoroughly investigated [43]. High-strength steels and non-ferrous metal alloys are the most widely used group of construction materials in modern cars. DP600 steel is used for production of the floor panels, front sub-frames, fenders, and B-pillars [23]. Meanwhile, Ti6Al4V is used to produce, among others, bumpers and components of car bodies. RSW is the basic technology for joining components in the automotive industry due to its high speed, the possibility of automating the joining process, and the high strength of the joints. The RSW of steel sheets is currently not a problem, but joining some dissimilar materials requires a special approach. As shown by the literature review, the RSW of Ti6Al4V with DP600 steel has not been thoroughly investigated. The development of joining dissimilar materials, including lightweight metals, by RSW is desirable due to the desire to reduce vehicle weight and the related reduction in fuel consumption. In this article, pilot studies were undertaken to improve the mechanical properties of RFW joints of Ti6Al4V to DP600 steel using Cu and Au interlayers.

A problem in joining titanium with steel is brittle Ti-Fe IMCs, which limit the achievement of adequate joint strength [44]. A method for overcoming this problem is to use a proper interlayer between joined sheets. Various approaches to the RSW of titanium and steel are specified in the literature using a Nb/Cu/Ni multilayer [45], Ya/V/Fe composite later, Cu interlayer [41], Ag interlayer [46], Ta interlayer [47], Ni interlayer [48], Nb interlayer [49], BAg45CuZn interlayer, and 60%Ni-Cu alloy interlayer [43]. A Cu interlayer can be considered as the most universal and inexpensive interlayer material that facilitates quality welds of dissimilar materials [50]. Copper exhibits an intrinsic absence of ductile-to-brittle transitional behavior [51]. Alternatively, an Au interlayer was used as part of the cognitive tests in this article. An Au interlayer may be used to prevent the formation of brittle intermetallic compounds, which reduce the joint strength. The process of depositing both the Cu and Au layers was carried out using the vacuum sputtering method. This is a controlled process of transferring atoms from the evaporation source to the object being coated. The obtained coating thickness was approximately 3 µm. The results obtained were compared with joints obtained by RSW without an interlayer. The effect of applying the Cu and Au interlayers on the resulting interfacial structure and microstructure and the selected properties of the joints were examined.

## 2. Experimental Procedure

### 2.1. Material

**Figure 1:**
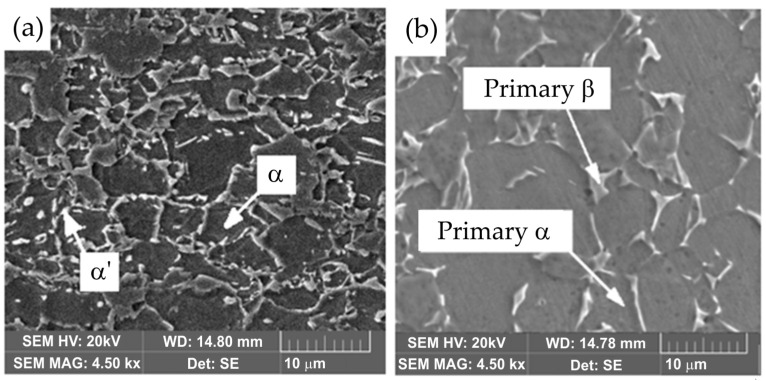
Microstructure of (**a**) DP600 steel and (**b**) Ti6Al4V titanium alloy.

The welding materials used in this experiment included Ti6Al4V titanium alloy and DP600 steel sheets. The welding test samples were cut into strips of 20 mm × 100 mm × 1.5 mm (Ti6Al4V) and 20 mm × 100 mm × 1.4 mm (DP600) using wire electrical discharge machining technology. The chemical composition and selected mechanical properties of DP600 steel are presented in Table 1 and Table 2, respectively. DP steel contains a small amount of alloying elements and has a relatively low carbon equivalent value of CEV = 0.23, so it can be easily welded with all alloy steels and ordinary structural steels using conventional arc welding methods. Dual-phase DP600 steel can be welded with similar linear energies as other high-strength steels. DP600 steel consists of a matrix in the form of soft ferrite grains (α) and hard inclusions of the second phase, usually martensite (α′), the amount of which is 5–30% (Figure 1). The soft phase has a positive effect on ductility. The hard phase is responsible for increasing the strength of the material and strengthening it during cold processing.

The second material used in the tests was the Ti6Al4V sheet. The chemical composition of this alloy is given in Table 3, and the selected mechanical properties are listed in Table 4. Ti6Al4V alloy is a two-phase α + β alloy. It is characterized by high strength and exceptional corrosion resistance. Two-phase α + β titanium alloys comprise the most numerous group of structural titanium alloys used. The two-phase structure is achieved by the appropriate amount of elements stabilizing the β phase (Mo, V, Ta, Nb, Fe, Mn, Co, Cu, and Cr) and aluminum, which dissolves well in both Ti-α and Ti-β. The α + β alloys have thermal treatment properties similar to steel. If quench hardening is carried out from the temperature of the existence of β phase, martensitic transformation causes hardening, and plasticity decreases. The transformation occurs within a specific temperature range, but this range decreases with a higher content of alloying elements. The resulting martensite is a supersaturated solid solution of elements in Ti-α and is designated α′. It is characterized by an acicular structure and a hexagonal crystal structure. After tempering the remaining β phase, high-quality alloys can be obtained as a result of precipitation hardening. However, in some alloys, a transition phase may occur as a result of precipitation hardening (especially with Cr, Mn, Zr, and Nb) and there is a risk of brittleness occurring.

**Figure 2:**
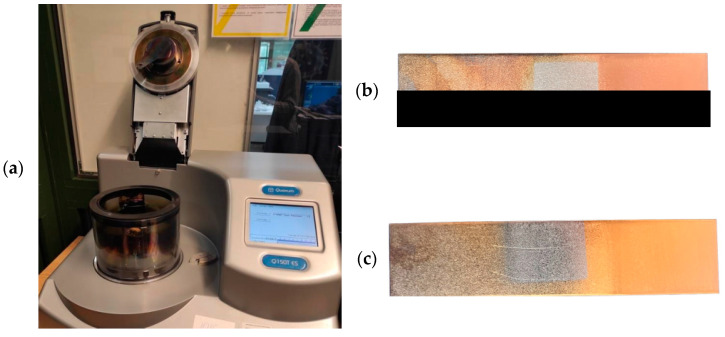
Sputter coating process: (**a**) Q150 turbo-pumped sputter coater; (**b**) Cu layer; (**c**) Au layer.

Copper (Cu) and gold (Au) were used as an interlayer. The samples were placed in a Q150 high-vacuum sputter coater (Quorum, San Jose, CA, USA) to deposit a thin layer of Cu and Au on the surface of the samples (Figure 2a). The interlayer was sprayed on strips of sheet metal, both on the Ti6Al4V titanium alloy and on DP600 steel (Figure 2b,c) in the area of joint overlap. Before the spray coating process, the surfaces of the materials were polished with sandpaper to a grit of 1000 # and then cleaned and degreased in acetone. Then, the strip sheets were placed in a Memmert air dryer (Memmert GmbH, Buchenbach, Germany) for 15 min at a temperature of 60 °C. Each sample was sprayed with the Cu and Au for 90 s. The current during spraying was 60 mA. Materials without the Cu and Au interlayers were also welded for comparison purposes.

### 2.2. Methodology of the Resistance Spot-Welding Process

**Figure 3:**
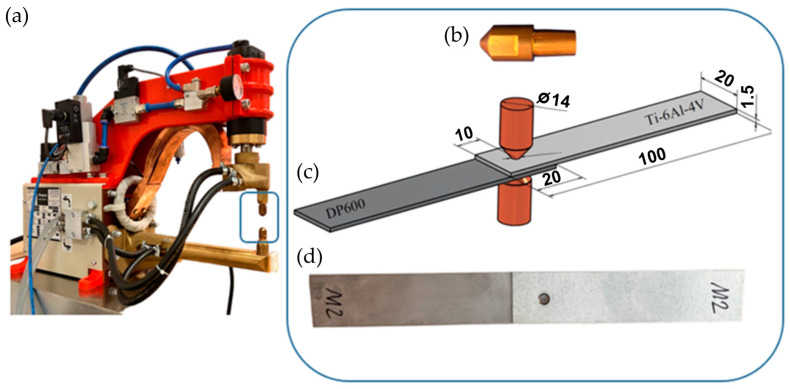
(**a**) ZPb-6 resistance spot-welder; (**b**) electrode; (**c**) lap RSW joint; (**d**) welded joint obtained.

**Figure 4:**
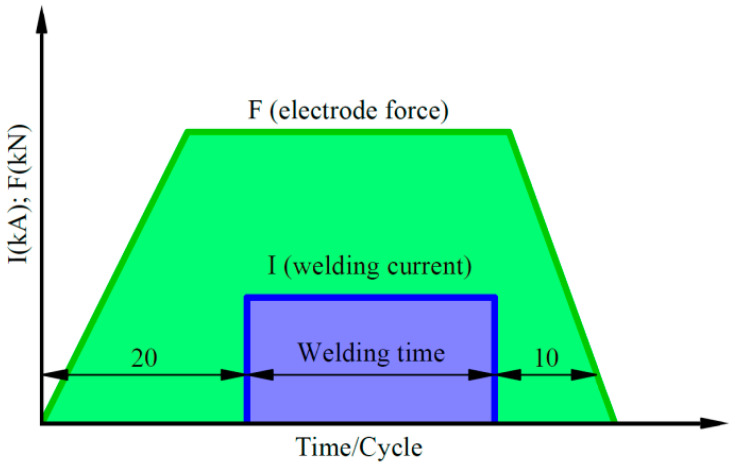
Variation of electrode force and welding current during RSW.

The RSW of lap joints was performed using a ZPb-6 resistance spot-welder (ASPA, Wroclaw, Poland) (Figure 3a). The tip diameter of the Cu alloy electrodes (Figure 3b) was 4 mm. The parameters of RSW, including the welding time, welding current, and electrode pressure force, are presented in Table 5. The values of the RSW process parameters were determined on the basis of preliminary research. The welding current is constant in each cycle. Dimensions of the specimens are presented in Figure 3c. The welded joint is shown in Figure 3d. Variation of electrode force and welding current during RSW is presented in Figure 4.

**Figure 5:**
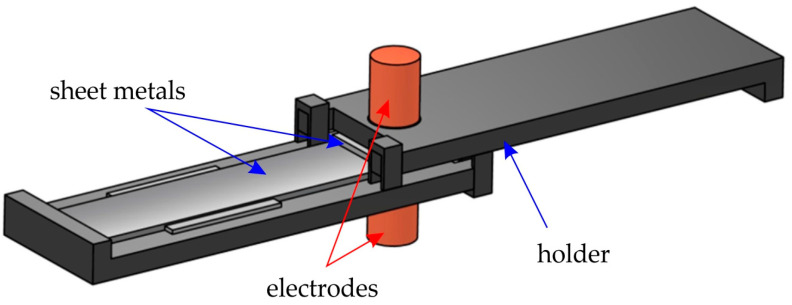
A holder for positioning samples in the welding process.

In order to ensure repeatable positioning of the sheet metal strips joined together during the welding process, a special holder was developed and manufactured (Figure 5). This holder allows obtaining the required parallelism of the joined strip samples, the required length of the overlap in the lap joint, and the central position of the weld in relation to the joined elements. The above requirements are necessary due to the subsequent strength analysis of the welded joints (especially in the tensile test).

After the welding process, the obtained joints were cut along the center of the spot-weld nugget, followed by polishing and etching in order to reveal the microstructure. The microstructure of welded joints was assessed using an optical microscope (OM) and a scanning microscope (SEM) on the cross-section of the obtained welded joints. SEM observations were performed on an MIRA3 scanning electron microscope (TESCAN, Brno, Czech Republic). Measurements of the hardness distribution of the welded joint in base material BM, heat affected zone HAZ, and weld zone WZ were made using the 60 M Vickers hardness tester (Qness, Mammelzen, Germany) in accordance with the ISO 6507 [52] standard. A load of 9.807 N (HV1) and a test time of 10 s were applied. Tensile-shear tests of welded joints were carried out on a Zwick/Roell Z100 testing machine in accordance with the EN ISO 6892-1 [53] standard. The test samples were cut on an electro-discharge cutting machine, thus avoiding thermal impact during the cutting process. Strip samples were cut from each joint along the center of the weld and were used to characterize the microstructure in the cross-section of the weld. Samples intended for metallographic examination were wet-polished with 220~2200 # grit paper and diamond paste (grain size 1–3 μm). The reagent used to etch the samples for approximately 15 s had the following chemical composition: 1 g of C_6_H_3_N_3_O_7_, 5 mL of HCl, and 100 mL of C_2_H_6_O. All experiments were repeated three times.

## 3. Results and Discussion

### 3.1. Hardness of the RSW Joint

**Figure 6:**
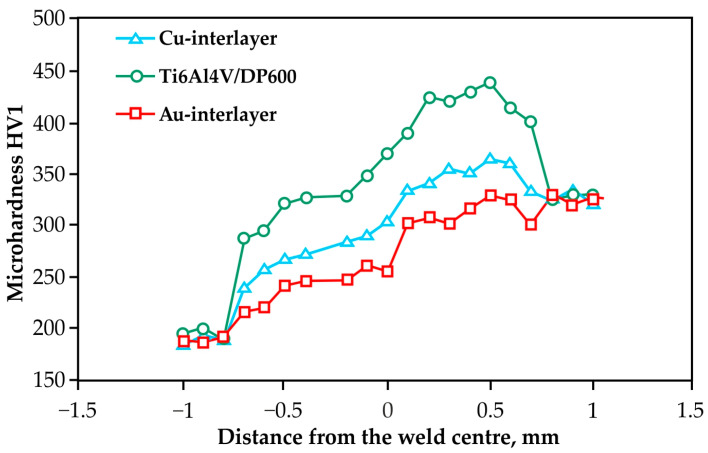
Vickers microhardness distribution in RSW joints.

The microhardness distributions in the welded joints obtained using the RSW method without an interlayer and with the use of an Cu or Au interlayer are shown in Figure 6. The distribution of microhardness in the RSW welded joints was qualitatively comparable for the joints both with and without an interlayer of Cu or Au. In the joints without the use of an interlayer, more areas of high hardness were found compared to the microhardness areas of welds fabricated with the presence of the Cu and Au interlayer. The highest hardness in the welded joint without an interlayer occurred in the weld nugget and was approximately 430 HV1, which can be attributed to the formation of intermetallic phases TiFe and TiFe_2_ in the welding process. This resulted in an increase in the microhardness of the weld nugget compared to base metals. The obtained microhardness in the case of joints welded with the Cu and Au interlayers was lower compared to joints produced without the presence of the interlayer. The tensile/shear strength of the resulting joint with the interlayer depended mainly on the type of interlayer used, which was directly related to the atomic diffusion between Ti6Al4V and DP600 during the welding process. This resulted in differences in microhardness values depending on the type of interlayer used.

In the welding process using a Cu interlayer, a significant reduction in the hardness of the joint was found. The highest hardness in the welded joint occurred in the weld nugget and amounted to approximately 350 HV1. This value is approximately 100 HV1 lower compared to the welded joint obtained without the use of an interlayer. When copper was used as an interlayer, the presence of metallic compounds produced in the welded joint was found. Reactions occurring between the melting temperature of CuTi phase particles and the eutectic transformation temperature resulted in the formation of intermetallic phases Ti_3_Cu_4_, Ti_2_Cu_3_, TiCu_2_, and TiCu_4_. Moreover, two equilibrium Cu_4_Ti phases may exist in CuTi_4_ phase-stable β and metastable α. The α phase is transformed into the β phase over time. In the welding process using an Au interlayer, the greatest decrease in the hardness value was observed in the obtained welded joint. The highest hardness in the welded joint occurred in the weld nugget and amounted to approximately 320 HV1. This value is lower by approximately 130 HV1 compared to the welded joint obtained without the use of an interlayer and lower by approximately 30 HV1 compared to the welded joint obtained with the use of a Cu interlayer. The effect of reducing hardness when using an interlayer with Au is due to the fact that some alloying elements, such as Fe, can lower the martensitic transformation temperature, and some elements, such as Au, increase the martensitic transformation temperature. The highest average microhardness in all joints was observed in the weld nugget. This is the result of the formation of an acicular microstructure of martensite in this zone.

**Figure 7:**
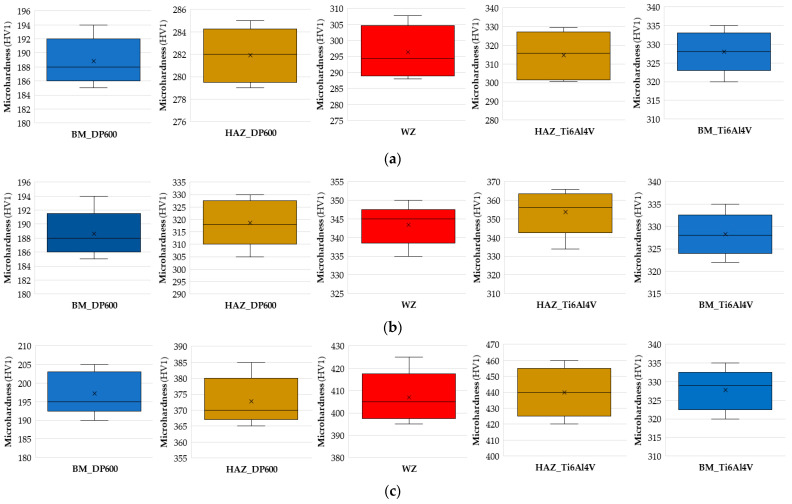
Distribution of microhardness values for RSW joints: (**a**) Au interlayer, (**b**) Cu interlayer, and (**c**) DP600-Ti6Al4V.

Figure 7 shows box-and-whisker plots [54,55] of the distribution of the obtained microhardness values depending on the intermediate layer used. The microhardness was listed for five areas of the welded joint: BM_DP600, HAZ_DP600, WZ, HAZ_Ti6Al4V, and BM_Ti6al4V. Below is the interpretation of the results obtained when a Cu interlayer was used. For BM_DP600, we can deduce that 25% of data has a value less than 186 HV1; similarly, the upper quartile represents 75% of the data. So, 75% of the data is less than 192 HV1. The maximum and minimum values obtained in the tests are 194 HV1 and 185 HV1, respectively. The bold black line in the box shows the median value of the data. The median is approximately 188 HV1. For HAZ_DP600, we can conclude that 25% of the data has a value lower than 279.5 HV1; similarly, the upper quartile represents 75% of the data. Therefore, 75% of the data is less than 284.2 HV1. The maximum and minimum values obtained in the tests are 285 HV1 and 279 HV1, respectively. The median for HAZ_DP600 is approximately 282 HV1. For WZ, we can conclude that 25% of the data has a value lower than 289 HV1. The upper quartile represents 75% of our data. The maximum and minimum values obtained in the tests are 307.8 HV1 and 288 HV1, respectively. For HAZ_Ti6Al4V, we can conclude that 25% of the data has a value lower than 301.5 HV1. The upper quartile for HAZ_Ti6Al4V represents 75% of the data. In this case, the median is approximately 315.9 HV1. For BM_Ti6al4V, 25% of the data has a value lower than 323 HV1; similarly, the upper quartile represents 75% of the data. It can be concluded that 75% of the data has less than 333 HV1, with a median of approximately 328 HV1.

### 3.2. Tensile Properties of RSW Joints

**Figure 8:**
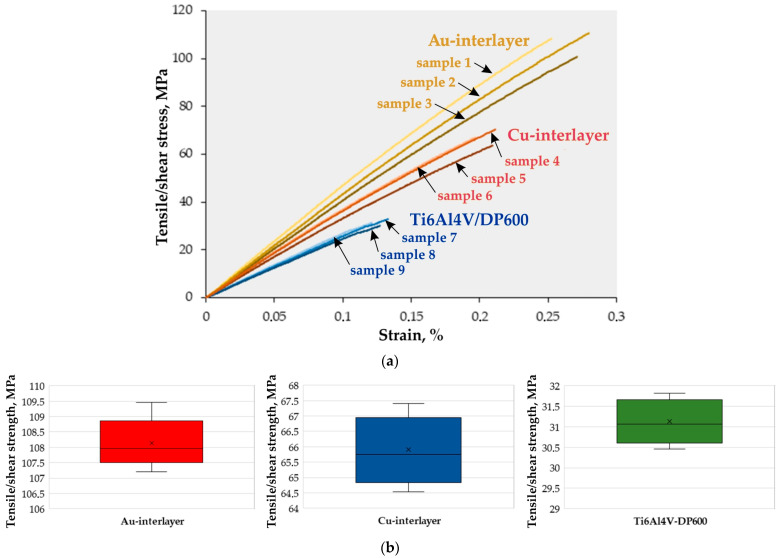
Tensile-shear test results (**a**) as a function of strain and (**b**) with scatter around the average tensile/shear strength value.

**Figure 9:**
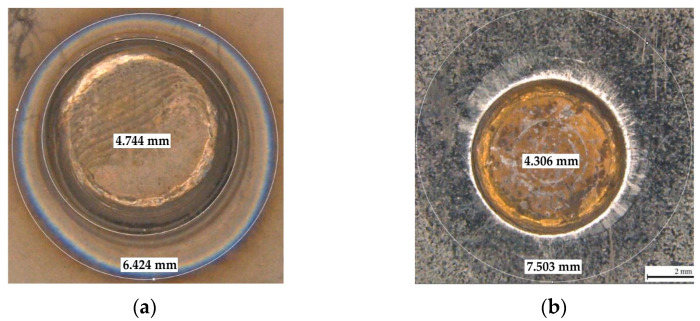
View of the obtained welded joint (**a**) from the Ti6Al4V material side; (**b**) from the DP600 material side.

The variation of the tensile-shear stress during stretching of the RSW joint is presented in Figure 8. Measurements of the diameter of the welds (Figure 9) obtained for the joints were carried out using an optical microscope both on the side of the Ti6Al4V material and on the side of the DP600 material. Measurements were made for all welded joints during the tests. The tensile-shear test results are presented for samples welded without an interlayer and the presence of the Cu and Au interlayers. The tensile-shear strength for samples welded without the presence of the interlayer was only 31.1 MPa.

Figure 8b shows a box-and-whisker plot of the distribution of the obtained stress values depending on the intermediate layer used. Looking at the graph for the Au interlayer case, we can immediately deduce that 25% of the data has a value less than 107.51 MPa; similarly, the upper quartile represents 75% of the data. The value of 75% of the data is less than 108.87 MPa. The maximum and minimum values obtained in the tests for joints with a Au interlayer are 109.46 MPa and 107.21 MPa, respectively. The bold black line in the boxes shows the median value of the data. In our example of an RSW joint with a Au interlayer, the median is approximately 107.96 MPa. Moreover, a positive skew was noticed in the stress distribution.

The tensile/shear strength of the RSW joints reached 65.5 MPa during RSW with the presence of a Cu interlayer, which is more than twice as much as in the case of welded joints without the presence of the interlayer. This is mainly due to the change in the microstructure in the contact zone caused by the addition of Cu and the precipitation of CuTi, Cu_3_Ti_2_, and TiCu phases, which partially replaced the TiFe metallic phase. During resistance welding with the presence of the Au interlayer, the tensile-shear strength of the joint reached 108 MPa, which is more than three times higher than in the case of welded joints without the use of an interlayer. This happens, as in the case of the Cu interlayer, as a result of a change in the microstructure in the contact zone caused by the addition of Au and the precipitation of TiAu, which replaced part of the TiFe metallic phase and significantly increased the martensitic transformation temperature. At the same time, for two types of welded joints consisting of interlayers of Cu and Au, no or fewer welding defects in the form of pores and cracks occurred. Therefore, interlayers significantly improved the quality of the RSW joints. In all welded joints, shear occurred at the spot-weld. The direct cause of this is the presence of the acicular α′ phase.

**Figure 10:**
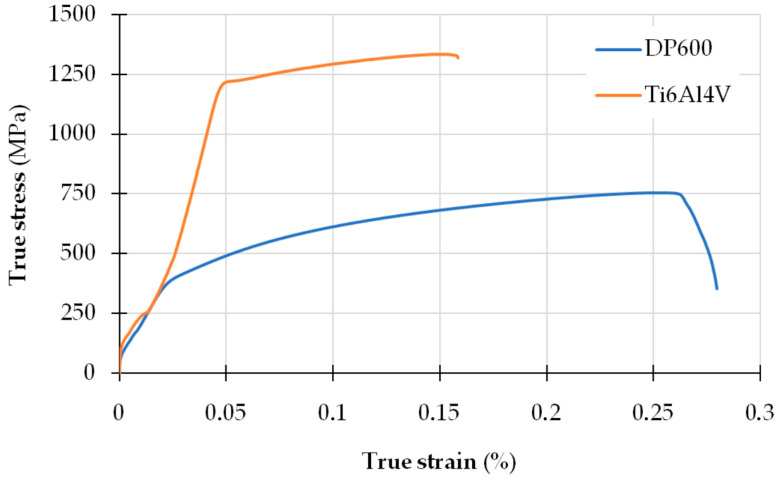
Stress–strain curves for Ti6Al4V titanium alloy and DP600 steel.

A static tensile test was performed on the base materials welded during the tests. Figure 10 shows true stress–strain curves.

Figure 8b shows a comparison of the tensile/shear strength [56] of the welded joint depending on the intermediate layer used. The following assumption was made for comparative purposes. The tensile/shear strength of the welded joints was treated as a case of uniaxial tension during a static tensile test of the base materials. As can be observed (Figure 8b and Figure 10), the highest shear strength occurs in the joint with the Au interlayer. The strength of this joint is only seven times lower than that of the base material (DP600). When using a Cu interlayer, an eleven-fold reduction in the tensile/shear strength value is observed. In a joint without an intermediate layer, a twenty-four-fold reduction in the strength value of this joint was recorded.

**Figure 11:**
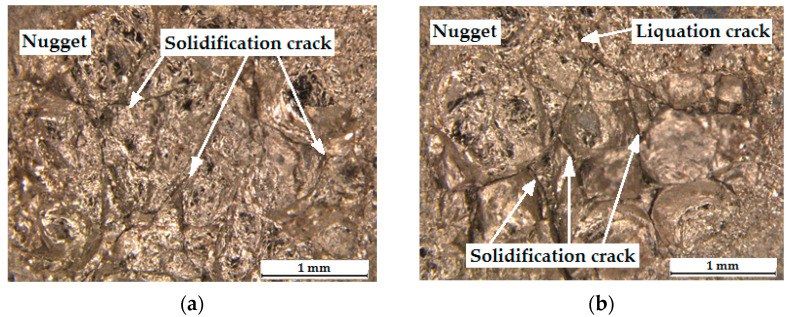
Surface of the RSW joint after shear test: (**a**) joint with Au interlayer; (**b**) joint with Cu interlayer.

Figure 11 shows views of the surface of the RSW joint after tensile/shear testing on the Ti6Al4V side with Au (Figure 11a) and Cu interlayers (Figure 11b). It can be seen that in both cases microcracks appear in the weld area, mainly near the center of the weld nugget. However, when an Au interlayer is used in the RSW process, a smaller number of cracks in the weld can be clearly observed. These weld imperfections can lead to a reduction in weld strength. Because the grains in the weld have different orientations relative to each other (dendritic structure), cracks are the result of the formation of IMCs and metal shrinkage during the solidification of the weld.

### 3.3. Macroscopic Characteristics of Welded Joints

**Figure 12:**
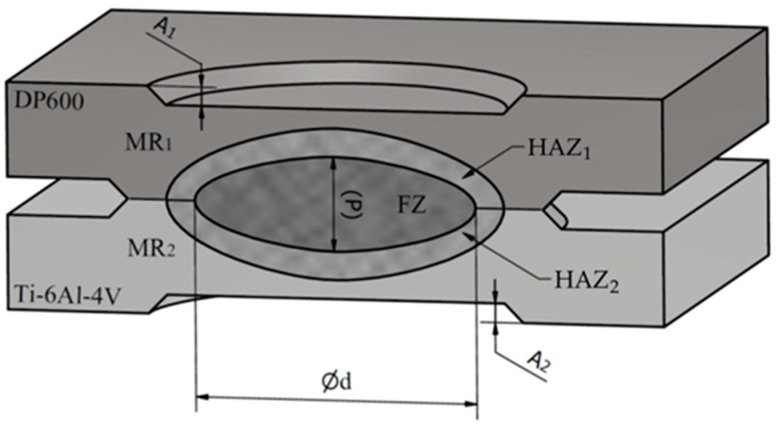
The shape of the obtained welded joint.

The Ti6Al4V/DP600 joint obtained in the RSW process has depressions (A_1_ and A_2_ in Figure 12), and the shape of the transverse shear surface of the welded joint is approximately elliptical (Figure 12).

In the cross-section of the RSW joint, specific areas can be distinguished, by which the resulting welded joint can be assessed. The boundary of the base metals (MR_1_ and MR_2_ in Figure 12) inside the molten nugget disappears, and the resulting weld nugget is homogeneous. The weld nugget zone moves towards the titanium alloy. Three different zones are marked in Figure 12. In the microstructure, the following zones are observed: the fusion zone (FZ), which melts and re-solidifies during the welding process and shows a dendritic structure; the heat-affected zone (HAZ), which does not melt but undergoes microstructural changes; and the base material (MR), which does not undergo microstructural changes. The resistivity of the Ti6Al4V titanium alloy is 0.42 μΩ·m, which is much higher than that of DP600 steel (0.13 μΩ·m). The shrinkage of the material volume as the weld solidifies causes stresses in the molten nugget. As a result, the metal in the molten nugget is not able to fill the cavity of molten material after solidification. As a result, pores are created in the welded joint. Because the coefficient of linear expansion of titanium alloy is different from that of steel, the shrinkage rate of the two materials is different when the temperature is lowered.

#### 3.3.1. Ti6Al4V/DP600 Joints without Interlayer

**Figure 13:**
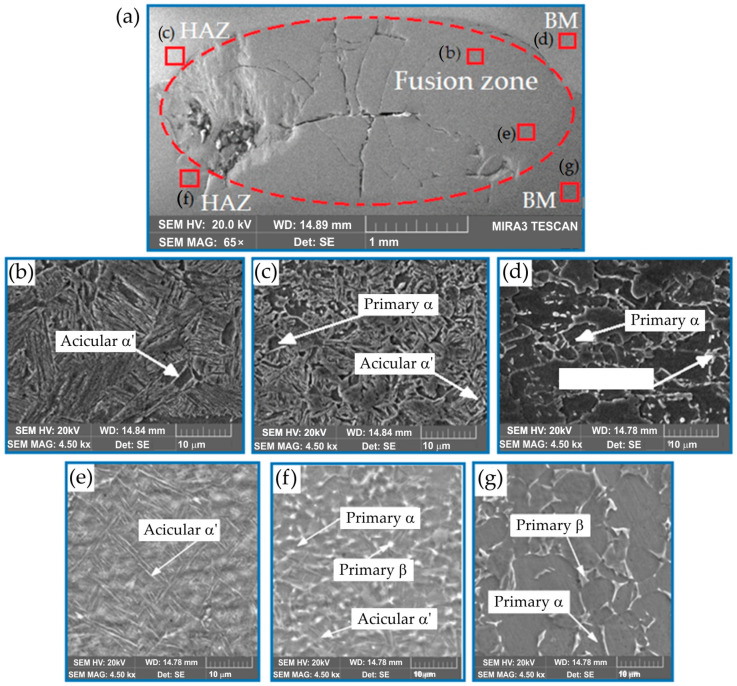
Morphology of the Ti6Al4V/DP600 spot-welded joint: (**a**) weld, (**b**) microstructure of the weld nugget on the DP600 steel side, (**c**) HAZ zone on the DP600 steel side, (**d**) microstructure of the DP600 base material, (**e**) microstructure of the weld nugget on the Ti6Al4V alloy side, (**f**) HAZ zone on the Ti6Al4V alloy side and (**g**) microstructure of the Ti6Al4V base material.

Figure 13 shows cracks in the welded Ti6Al4V/DP600 joint. The larger the molten pool, the greater the tensile stresses in the weld nugget that occur during solidification. In the absence of an interlayer, the microstructure of the Ti6Al4V/DP600 spot-welded joint is determined, as shown in Figure 13, and it can be seen that the welded joint is composed of macroscopic cracks and pores. During the solidification process, the metal in the molten pool is subjected to local stresses and deformations as a result of contraction in volume. As a result, pores are formed in the molten nugget during the solidification process. The shrinkage rate of both materials is different when the weld crystallizes. Therefore, high internal stresses arise in the welded joint, which causes cracks and pores to appear.

**Figure 14:**
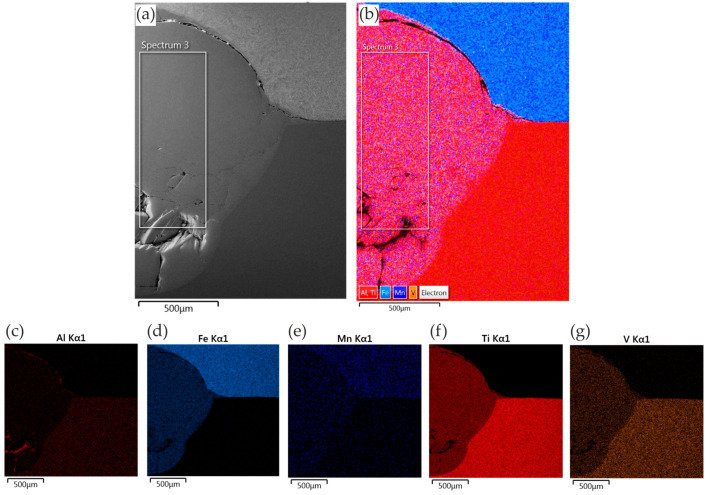
EDS mapping of elements in the RSW joint without presence of interlayer: (**a**) SEM micrograph; (**b**) element distribution; and distribution of specific elements: (**c**) Al; (**d**) Fe; (**e**) Mn; (**f**) Ti; and (**g**) V.

To more precisely describe the microstructure of the joints, the morphology of the samples was analyzed both on the Ti6Al4V alloy side and on the DP600 steel side using SEM. An example of the morphology of the resulting welded joint is shown in Figure 14. As shown in Figure 14a, typical brittle cracks and pores were formed in the RSW joint (weld nugget), which resulted in a joint with lower strength properties. The weld nugget (Figure 14b,e) has a typical acicular structure, with clearly outlined areas between the acicular martensite (α′). Based on the analysis of the phase equilibrium system, it can be assumed that dendrites with a chemical composition richer in titanium originally crystallized from the liquid. Further, during cooling, a eutectoid transformation occurred, resulting in the formation of a mixture of the α-Ti phase and the FeTi intermetallic phase. Observation of weld microstructure using scanning electron microscopy revealed the complex microstructure of dendrite cores, where the α-Ti phase was observed in the form of plates in the Widmanstatten’s system. As the observation site moved away from the center of the weld nugget, a different morphology of the microstructure was observed. Right next to the HAZ boundary, there is a narrow zone with a lamellar structure (Figure 14c,f). This indicates that it is a base metal that has been partially melted but has not been mixed with adjacent material. The martensite α′ with acicular structure formed in the joint is a supersaturated solid solution of elements in Ti-α. It has a hexagonal crystal structure. During spot-welding of Ti6Al4V/DP600, FeTi intermetallic compounds with high hardness and brittleness are formed in the weld nuggets. The RSW joint mainly consists of a TiFe_2_ + α-Fe eutectic structure near the DP600 steel side and a TiFe + α-Ti eutectic structure on the Ti6Al4V alloy side. The presented results indicate that layers of TiFe and TiFe_2_ intermetallic compounds are formed on welded joints. For this reason, brittle fracture and welding pores appeared in welded joints.

#### 3.3.2. Ti6Al4V/DP600 Joints with Cu Interlayer

**Figure 15:**
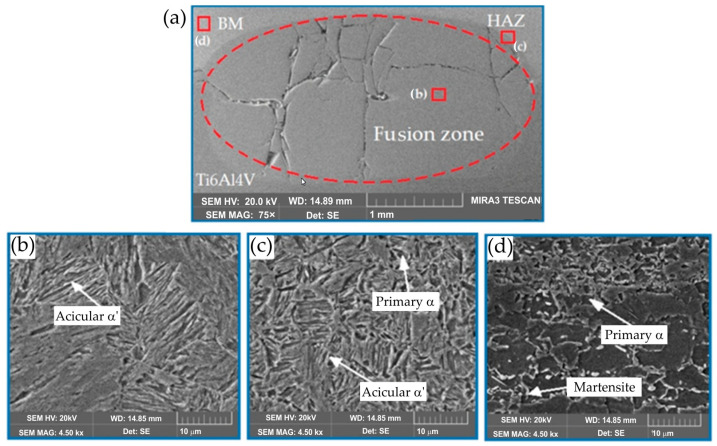
Morphology of the Ti6Al4V/DP600 spot-welding joint with a Cu interlayer: (**a**) weld, (**b**) microstructure of the weld nugget, (**c**) HAZ on the DP600 side, (**d**) microstructure of the DP600 base material.

The morphology of the resulting welded joint containing the Cu interlayer is shown in Figure 15. Typical brittle fractures were formed in the RSW joint (weld nugget), as in the case of a joint made without an interlayer. However, no pores were observed, which drastically reduce the strength properties of the welded joint. The weld nugget (Figure 15b) also has a typical acicular microstructure with clearly outlined areas between the acicular martensite (α′). Observation using scanning electron microscopy revealed the complex microstructure of dendrite cores, where the α-Ti phase was observed in the form of plates. In the HAZ, there is a narrow zone with a lamellar microstructure (Figure 15c). The martensite formed in the joint is a supersaturated solid solution of elements in Ti-α (α′). During spot-welding of Ti6Al4V/Cu/DP600, FeTi IMCs are formed in welded joints, but in smaller amounts than in the case of a welded joint without an interlayer. The joint mainly consists of a TiFe_2_ + α-Fe eutectic microstructure near the DP600 steel side and a TiFe + α-Ti eutectic microstructure on the Ti6Al4V alloy side.

**Figure 16:**
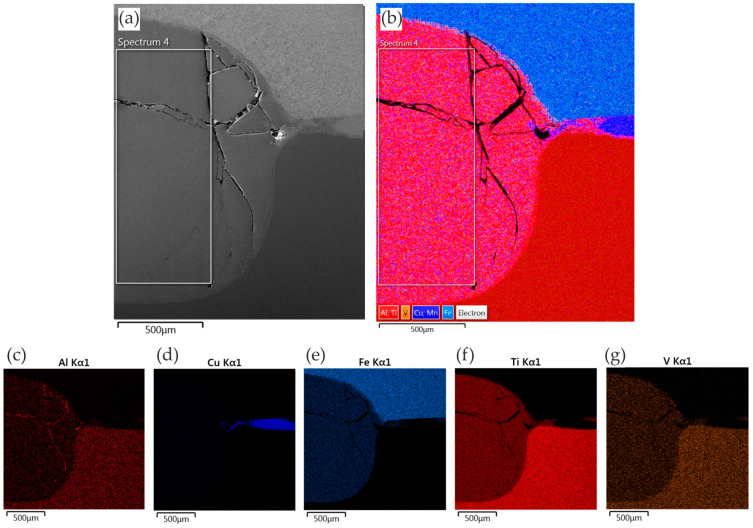
EDS mapping of elements in the RSW joint with the presence of a Cu interlayer: (**a**) SEM micrograph; (**b**) element distribution; and distribution of specific elements: (**c**) Al; (**d**) Cu; (**e**) Fe; (**f**) Ti; and (**g**) V.

Figure 16 shows the results of SEM-EDS analysis of the joints consisting of a Cu interlayer. The elements Ti, Fe, Al, V, Mn, and Cu were disclosed in the joints. Elemental transfer occurred in the contact zone as a result of the heat (Figure 16). The results of the EDS mapping show that the degree of diffusion of the Ti was greater than that of the elements Fe, Al, V, and Mn. This is due to the fact that the diffusion coefficient of the elements Fe, Al, V, and Mn in the range of welding temperatures is lower. Few instances of the Ti element dispersed on the DP600 steel side were observed (Figure 16f), and no Fe element was dispersed on the Ti6Al4V alloy side (Figure 16e). The existence of the interlayer hindering the mutual diffusion of Fe and Ti is attributed to the fact that the interfacial reaction of TiFe in the welding process without an interlayer was transformed into the interfacial reactions of TiCu and FeCu in the resistance welding process with the Cu interlayer. This process additionally proved that the FeTi intermetallic phase was partially blocked in the RSW process with the presence of the Cu interlayer.

**Figure 17:**
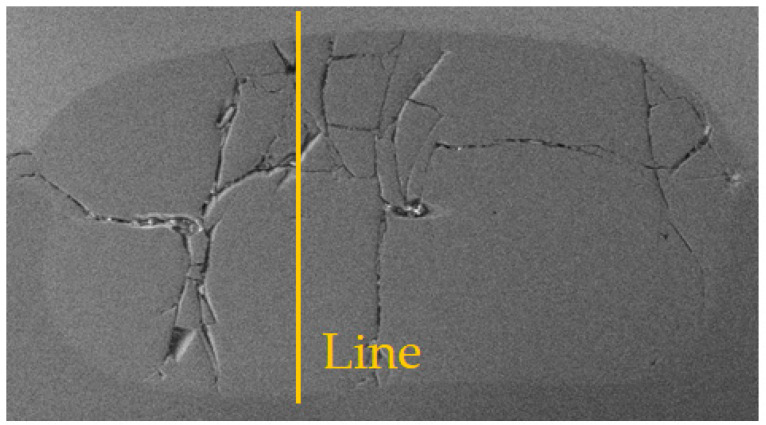
The cross-section of the Ti6Al4V/DP600 weld.

Figure 17 shows an optical micrograph of a weld of Ti6Al4V/DP600 materials taken by backscattered electron (BSE) imaging in the cross-section of the weld. As can be seen in Figure 17, the lower layer of titanium alloy is darker in color, and the upper layer of DP600 steel is lighter in color. The color of the weld appears as a mixture of alternating light and darker colors, which indicates the mixing of both materials at high temperature in the molten core of the weld and the mutual diffusion and migration of elements at the interface of the materials during the welding process. Titanium alloy and steel were melted in the area of action of the RSW electrode. 

**Figure 18:**
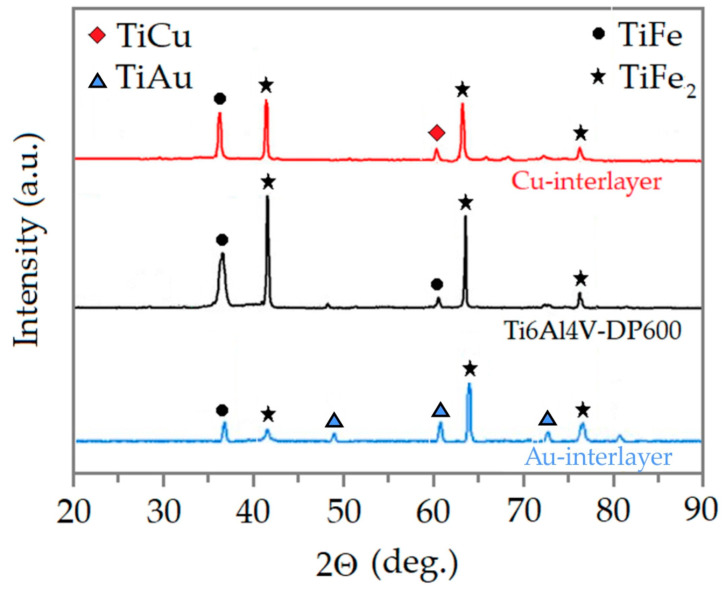
XRD results the RSW joints.

For each RSW joint with a Cu or Au interlayer and without an interlayer, XRD analysis (Figure 18) was performed in the area marked with a line in Figure 17. When using a Cu or Au interlayer, a small number of Ti elements were dispersed in the DP600 steel and a few Fe elements were dispersed in the Ti6Al4V alloy. In other words, the introduction of an interlayer hindered the mutual diffusion of Ti and Fe elements. The formation of the TiFe and TiFe_2_ metallic phase in the RSW process was probably converted to TiCu and TiAu interfacial compounds in the RSW process using Cu and Au interlayers. This phenomenon also further proved that the IMCs of TiFe and TiFe_2_ were effectively suppressed by using a Cu or Au interlayer in the RSW process.

#### 3.3.3. Ti6Al4V/DP600 Joints with Au Interlayer

**Figure 19:**
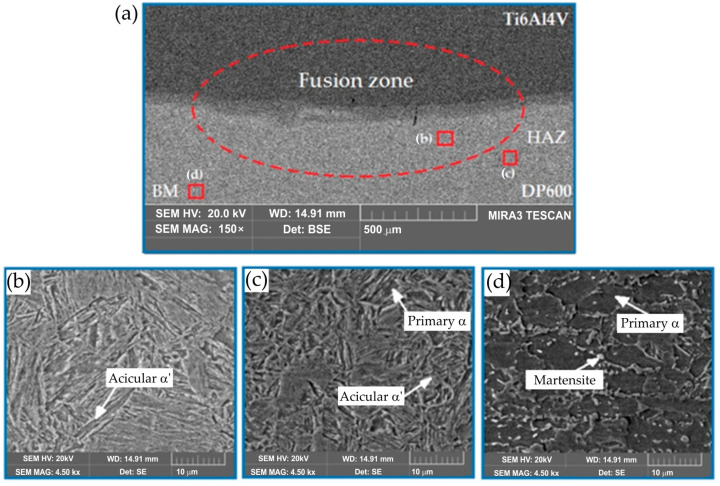
Morphology of the Ti6Al4V/DP600 spot-welding joint with a Au interlayer: (**a**) weld, (**b**) microstructure of the weld nugget, (**c**) HAZ on the DP600 side, (**d**) microstructure of the DP600 base material.

**Figure 20:**
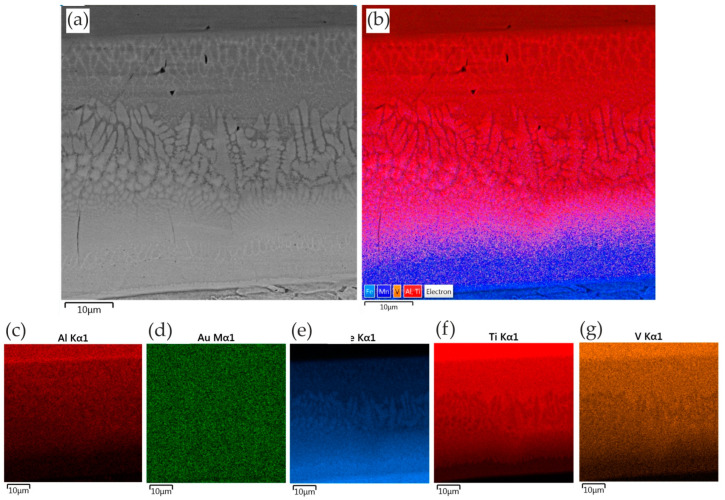
EDS mapping of elements in the RSW joint with the presence of the Au interlayer: (**a**) SEM micrograph; (**b**) element distribution; and distribution of specific elements: (**c**) Al; (**d**) Au; (**e**) Fe; (**f**) Ti; and (**g**) V.

The morphology of the weld after adding the Au interlayer is shown in Figure 19. It was found that the DP600 steel adjacent to the weld joint forms a concave surface. Due to the difference in the thermal conductivity of Ti6Al4V and DP600 materials, a large temperature gradient is created along the direction of the plate during the welding process. EDS mapping analysis showed that the active element Ti diffused from the Ti6Al4V alloy substrate and spread throughout the weld nugget. Melted Au diffused into the Ti6Al4V alloy substrate. Figure 20 shows the microstructure of the Ti6Al4V/Au/DP600 steel joint at a high magnification. Characteristic areas adjacent to titanium are shown in Figure 20c–g. According to the EDS results and the Ti–Au phase equilibrium system shown in [57], the reaction layers formed from the titanium substrate on the Al_2_O_3_ substrate were TiAu, TiAu_2_, Ti_3_Au, and TiAu_4_ phases and a Au phase containing TiAu_4_ particles. The hardness of TiAu and TiAu_4_ is quite similar, about 250 HV, and close to titanium hardness, whereas the hardness of Ti_3_Au is ~800 HV [58]. The hardness of Ti_3_Au is four times that of pure Ti and most steel alloys [59]. Due to high hardness, these IMCs exhibit brightness [60]. Karimia and Cattin [61] and Chiu et al. [62] reported that the brittle Ti_3_Au intermetallic compound in some specific amount deteriorates the ductility of alloys.

To explain the mechanism of formation of the typical TiAu interfacial microstructure in the Ti6Al4V/Au/DP600 joint, the TiAu microstructure was analyzed using the Ti–Au phase equilibrium system presented in [57]. The complex morphology of the interfacial microstructure and the distribution of various IMCs formed are dependent on the welding process temperature. The RSW process of Ti6Al4V/Au/DP600 can be described in a manner consistent with the Ti–Au phase equilibrium system. 

The Au melts when the temperature exceeds 1064 °C. As the concentration of Ti in the liquid phase increased, titanium reacted with molten Au to form the metallic phase TiAu_4_ through the peritectic reaction. In this way, a continuous TiAu_4_ layer was formed in the weld, which inhibited the diffusion of Au and Ti. Due to the decreasing concentration gradient from Ti6Al4V to the TiAu4 layer, new intermetallic phases, such as Ti_3_Au and TiAu, were formed. They were formed between the Ti6Al4V alloy and the TiAu_4_ layer.

## 4. Conclusions

This article attempts to determine the impact of the use of a Cu and Au interlayer on the mechanical properties and microstructure of Ti6Al4V/DP600 RSW joints. The main conclusions are as follows:

- A typical fracture mode of Ti6Al4V/DP600 joints made without the use of an interlayer is brittle fracture. The fracture surface of the resulting joints consist mainly of the intermetallic phase FeTi and Fe_2_Ti. The microstructure of the Ti6Al4V/Au/DP600 joint contained intermetallic phases Ti_3_Au, TiAu, and TiAu_4_, while the microstructure of the Ti6Al4V/Cu/DP600 joint contained intermetallic phases TiCu and FeCu.
- During the solidification process, the metal is unable to fill the molten cavity after crystallization due to local stresses and strains. As a result, pores are formed in the welded joint. This is because the coefficient of linear expansion of titanium alloy is different from that of DP600 steel; the shrinkage rate of the two materials is different when the temperature is lowered. The interlayers consisting of high-ductility metals (Cu and Au) reduce the number of cracks and pores and thus significantly improve the tensile/shear strength of the joints.
- Based on the tensile/shear strength of Ti6Al4V/DP600 joints without an interlayer and Ti6Al4V/DP600 joints with the presence of Cu and Au interlayers, it can be concluded that the Cu and Au interlayers cause a significant increase in the strength of the RSW joints. For the joint with the Cu interlayer, the maximum tensile/shear strength reaches 65.8 MPa; this is 100% higher than that of a joint obtained without an interlayer. The joint produced with the presence of the Au interlayer exhibits a tensile/shear strength of 108.3 MPa; this is 300% higher than that of the joint obtained without the interlayer. This is mainly due to the change in microstructure in the contact zone caused by the addition of Cu or Au and the precipitation of metallic phases based on Cu and Au, which replaced part of the TiFe metallic phase.

Due to the beneficial effect of the use of a Au interlayer on the mechanical properties of the RSW joints, research will be continued in the future. The influence of welding process parameters on the microstructure and selected mechanical properties (hardness, tensile properties) of Ti6Al4V/Au/DP600 joints will be determined.

## Figures and Tables

**Table 1:** Chemical constitution of DP600 material.

Element	C	Si	Mn	P	Cr	Al
Content (wt.%)	0.11	0.26	1.46	0.02	0.45	0.01

**Table 2:** Selected mechanical properties of DP600 steel.

R_m_, MPa	R_p0_._2_, MPa	A_50_, %	HV1
645	420	14	192

**Table 3:** Chemical constitution of Ti6Al4V material.

Element	Al	O	Si	V	Fe	C	N	Ti
Content (wt.%)	6.23	0.20	0.17	3.94	0.32	0.02	0.01	Balance

**Table 4:** Selected mechanical properties of Ti6Al4V titanium alloy.

R_m_, MPa	R_p0_._2_, MPa	A_50_, %	HV0.5
865	817	15	320

**Table 5:** Experimental conditions of RSW process.

Welding Current, kA	Welding Time, s	Electrode Force, kN
7	0.5	3

## Data Availability

The original contributions presented in the study are included in the article, further inquiries can be directed to the corresponding author.
